# Association between the serum uric acid-to-HDL-cholesterol ratio and female infertility: The mediating role of BMI

**DOI:** 10.1097/MD.0000000000047631

**Published:** 2026-02-13

**Authors:** Jia-Yi Zhao, Lin Zhang, Ming-Xuan Du, Quan Yang, Meng-Hui Hong

**Affiliations:** a Department of Clinical Laboratory, The Affiliated Foshan Women and Children Hospital, Guangdong Medical University, Foshan, Guangdong, China.

**Keywords:** BMI, female infertility, HDL cholesterol, uric acid, uric acid-to-HDL-cholesterol ratio (UHR)

## Abstract

The uric acid-to-high-density lipoprotein-cholesterol ratio (UHR) reflects systemic oxidative stress and metabolic imbalance, both of which have been implicated in infertility. This study aimed to examine the association between UHR and female infertility and to evaluate the mediating role of body mass index (BMI). In this cross-sectional study, data from 2570 women aged 18 to 45 years were obtained from the National Health and Nutrition Examination Survey (NHANES, 2011–2018). Fertility status was determined from the Reproductive Health Questionnaire. UHR was Box-Cox transformed, and its association with infertility was assessed using logistic regression, spline curve fitting, subgroup analyses, and causal mediation analysis. The prevalence of infertility was 13.54% (348/2570). Logistic regression analysis was used to explore the relationship between the Box-Cox transformation UHR and infertility, with an OR of 1.834 (95% CI: 1.244–2.702). Women in the highest UHR quartile had nearly twice the odds of infertility compared with those in the lowest quartile (OR: 1.989, 95% CI: 1.147–3.452). A linear dose–response relationship was observed. Mediation analysis indicated that BMI partially mediated the association between UHR and infertility, accounting for 38.4% of the total effect; the indirect effect was statistically significant (*P* < .001). Elevated UHR, a biomarker reflecting oxidative and metabolic stress, is associated with an increased risk of female infertility, and this relationship is partially mediated by BMI. Monitoring uric acid and high-density lipoprotein-cholesterol levels, alongside preconception BMI management, may improve reproductive outcomes. Further prospective studies are warranted to confirm causality.

## 1. Introduction

Infertility, defined as the inability to conceive after ≥12 months of regular unprotected intercourse,^[1]^ affects ~15% of reproductive-aged couples globally.^[2]^ Its rising prevalence, particularly in low-resource settings,^[3]^ extends beyond individual quality of life and poses significant public health burdens, including psychological distress, socioeconomic strain, and marital discord.^[4,5]^ Recognized as a societal disorder by the World Health Organization (WHO) and a priority by the Centers for Disease Control and Prevention, infertility warrants targeted preventive and clinical interventions.

Among modifiable risk factors, abnormal body mass index (BMI; underweight: BMI < 18.5 kg/m^2^; overweight/obesity: ≥25 kg/m^2^) is strongly linked to impaired fecundability. Obesity (BMI ≥ 30 kg/m^2^) elevates the risk of ovulatory infertility by 2- to 3-fold compared to women with a normal BMI (18.5–24.9 kg/m^2^).^[6]^ Female factors contribute to 33% to 41% of infertility cases,^[1]^ primarily due to ovulatory dysfunction and tubal pathology.^^[7,8]^^ Emerging evidence suggests that oxidative stress (OS) and inflammation may disrupt oocyte quality, folliculogenesis, and endometrial receptivity.^[9–12]^ Additionally, lifestyle factors (e.g., diet) influence reproductive outcomes.^[13]^

Uric acid (UA), the end product of purine metabolism, reflects systemic OS because it is generated through the xanthine oxidase pathway, which produces reactive oxygen species.^[14–16]^ In addition to OS, UA influences lipid/glucose metabolism and inflammation,^[17,18]^ with hyperuricemia associated with metabolic syndrome, insulin resistance, and cardiovascular disease.^[19]^ Notably, elevated UA levels correlate with a higher risk of female infertility. In contrast, high-density lipoprotein cholesterol (HDL-C) reduces atherosclerosis and OS.^[20]^ UA and HDL-C exhibit antagonistic roles in inflammation; UA and HDL-C play opposing roles in inflammation: UA impairs HDL-C function,^[21]^ whereas HDL-C depletion exacerbates metabolic dysfunction.^[22]^ Therefore, the uric acid-to-high-density lipoprotein-cholesterol ratio (UHR) may more accurately reflect oxidative and inflammatory status than either marker alone.

UHR has emerged as a biomarker for metabolic and inflammatory conditions (e.g., diabetes, chronic kidney disease),^[23–25]^ with preliminary evidence suggesting its utility in predicting female infertility. However, mechanistic insights remain scarce, particularly regarding BMI’s potential mediating role. This cross-sectional study aims to investigate the association between UHR and female infertility, with a focus on clarifying their relationship and exploring the contribution of BMI.

## 2. Materials and methods

### 2.1. Data source and study population

The data for this study were obtained from the National Health and Nutrition Examination Survey (NHANES), conducted by National Center for Health Statistics (NCHS). NHANES has been used to monitor and assess the nutritional and health status of the US population since the 1960s. This nationwide cohort survey involves approximately 5000 participants each year and plays a pivotal role in shaping health policy decisions.^[26]^ The survey includes interviews to collect demographic, socioeconomic, dietary and health information, and physical examinations and laboratory tests are conducted at mobile examination centers. NHANES is a publicly available dataset approved by the NCHS Ethics Review Board, with all participants providing written informed consent for data collection.

**Figure 1.**
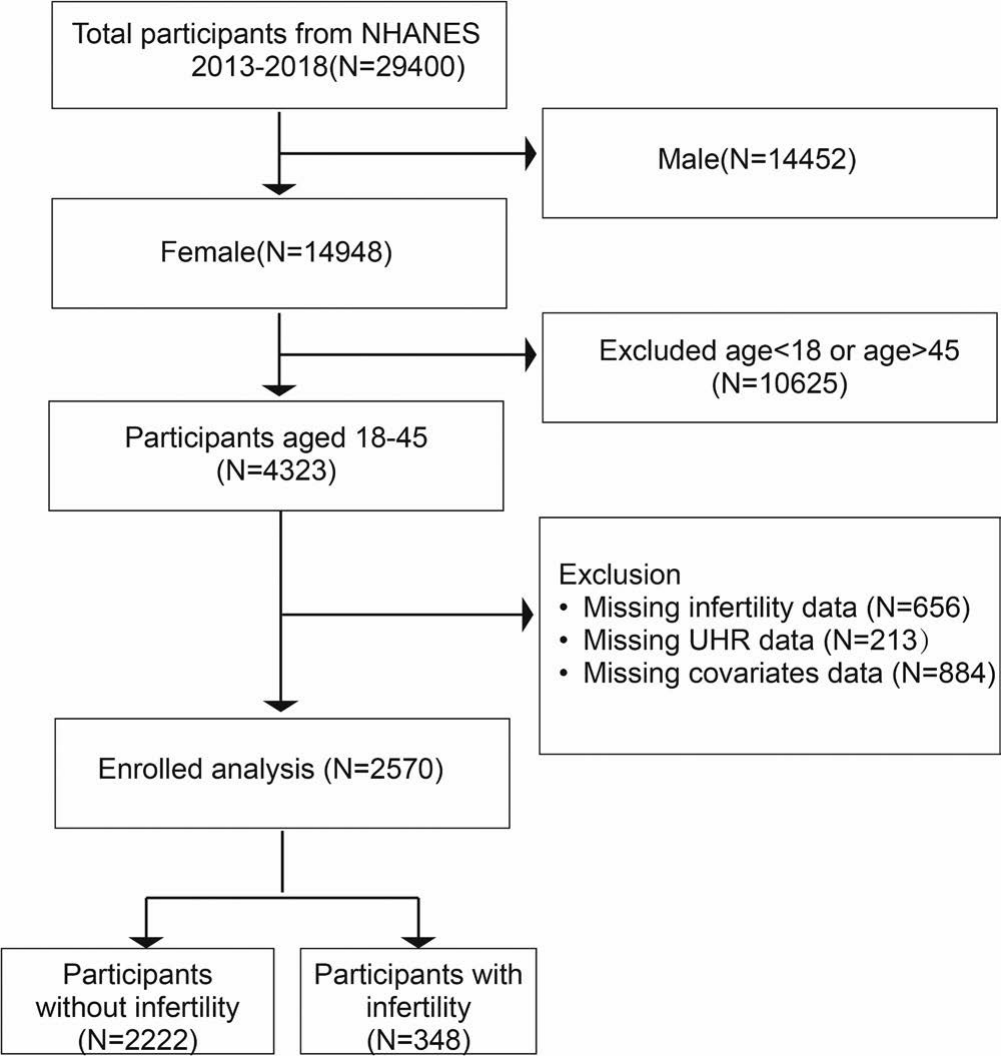
Screening flow chart of study participants. NHANES = National Health and Nutrition Examination Survey.

We included participants from the NHANES data collected between 2013 and 2018. A total of 29,400 participants were screened during this period. Initially, male participants (n = 14,452) were excluded. Women outside the reproductive age range (over 45 years or under 18 years, n = 10,625) were also excluded. Additionally, female participants with missing data for infertility (n = 656), UHR (n = 213), or covariates (n = 884) were removed from the analysis. Ultimately, 2570 eligible female participants were included in the study (Fig. 1).

### 2.2. Study variables

UHR was calculated as the ratio of UA (mg/dL) to HDL-C (mg/dL). Blood samples were collected from participants in a fasting state, and serum samples were separated within 1hour post-collection. NHANES provided standardized methods for each laboratory test item, including measurements of UA, HDL-C, Triglycerides, total cholesterol, albumin, serum creatinine.

Infertility status was diagnosed using the Reproductive Health Questionnaire (RHQ074): “Have you ever attempted to become pregnant over a period of at least a year without becoming pregnant?” Women who answered “yes” were classified as infertile, and this method has been widely used in other infertility studies. Potential confounders were generated from the NHANES health questionnaire, including age, ethnicity, education level, family poverty–income ratio, marital status, smoking status, and more.

### 2.3. Statistical analysis

Following NCHS guidelines, appropriate survey weights were applied in all analyses (https://www.cdc.gov/nchs/nhanes/index.html). Continuous variables were summarized as means ± standard deviations, and categorical variables were presented as frequencies (%). UHR values were transformed using the Box-Cox method due to non-normal distribution. Comparisons between groups were made using *t*-tests for continuous variables and the chi-square test for categorical variables.

Logistic regression analyses were performed to evaluate the association between UHR and infertility. Both univariate and multivariate models were constructed. The multivariate models were adjusted for the following potential confounders: age, race, education level, marital status, family poverty–income ratio, triglycerides, total cholesterol, albumin, creatinine, smoking status, alcohol use, hypertension, and diabetes.

Sensitivity analyses were conducted by categorizing UHR into quartiles and excluding participants with hypertension or diabetes to assess the robustness of the findings. Subgroup analyses were performed by stratifying participants according to age (<35 vs ≥35 years), race/ethnicity, alcohol use, smoking status, diabetes, hypertension, family income, pelvic inflammatory disease history, menstrual regularity, contraceptive use, and BMI category.

The dose–response relationship between UHR and infertility was assessed using restricted cubic splineregression with 3 knots (R “rms” package). Nonlinearity was formally tested using a likelihood ratio test comparing models with and without spline terms.

The potential mediating role of BMI was examined using a bootstrap-based causal mediation analysis with 5000 resamples, conducted in R version 4.2.1 (R Foundation for Statistical Computing in Vienna, Austria, http://www.r-project.org) using the “mediation” package. The analysis followed the Baron and Kenny causal steps framework, estimating total, direct, and indirect effects, along with the proportion mediated. Statistical significance was determined using bias-corrected 95% confidence intervals, with 2-sided *P* < .05 considered significant.

## 3. Results

### 3.1. Baseline characteristics of participants

Figure 1 shows the inclusion and exclusion criteria for the study participants. Among the 2570 eligible participants, 348 women were diagnosed with infertility, yielding a crude prevalence of 13.54% (348/2570). The baseline characteristics of the study population are presented in Table 1. Compared to non-infertile women, infertile women were older (35.36 years vs 32.00 years), more likely to be married or living with a partner (74.57% vs 57.09%), had higher BMI (≥30, 53.05% vs 37.84%), and had a higher o prevalence of hypertension (25.24% vs 13.73%), pelvic inflammatory disease history (9.46% vs 3.64%), and history of birth control pill use (80.42% vs 74.20%). Infertile women also had higher levels of UA (4.81 mg/dL vs 4.53 mg/dL), triglycerides (1.46 mmol/L vs 1.29 mmol/L) and total cholesterol (4.80 mmol/L vs 4.64 mmol/L). Furthermore, infertile women were more likely to have higher Box-Cox-transformed UHR. When UHR was categorized into quartiles, there were statistically significant differences between the infertile and non-infertile groups. However, no significant differences were observed between the 2 groups in terms of race, family income, education level, alcohol consumption, diabetes prevalence, smoking status, HDL-C levels, albumin, or creatinine.

**Table 1:** Weighted comparison in basic characteristics.

Characteristics	Total	Without infertility	Infertility	*P* value
Age (yr)	32.49 ± 0.23	32.00 ± 0.23	35.36 ± 0.53	<.0001
Age				<.001
<35	60.99 (55.66, 66.32)	63.20 (60.58, 65.82)	47.99 (40.67, 55.32)	
≥35	39.01 (34.84, 43.18)	36.80 (34.18, 39.42)	52.01 (44.68, 59.33)	
Race				.06
Non-Hispanic White	58.46 (50.15, 66.78)	57.40 (52.72, 62.09)	64.71 (57.19, 72.22)	
Non-Hispanic Black	12.39 (9.94, 14.83)	12.49 (9.66, 15.31)	11.80 (8.44, 15.16)	
Mexican American	11.35 (8.58, 14.12)	11.58 (8.74, 14.42)	10.02 (5.57, 14.47)	
Other Hispanic	7.21 (5.84, 8.57)	7.67 (6.21, 9.13)	4.47 (2.19, 6.76)	
Other race	10.59 (8.92, 12.26)	10.86 (9.16, 12.56)	9.00 (6.13, 11.88)	
Ratio of family income to poverty				.25
≤1.5	31.50 (28.81, 34.19)	32.28 (29.25, 35.30)	26.90 (21.35, 32.45)	
1.5–3.5	33.17 (29.61, 36.74)	32.79 (30.15, 35.43)	35.44 (29.59, 41.29)	
>3.5	35.33 (29.88, 40.77)	34.93 (31.26, 38.60)	37.66 (30.21, 45.12)	
Education level				.64
More than high school	71.76 (64.24, 79.29)	71.71 (68.02, 75.40)	72.09 (65.27, 78.91)	
High school	25.47 (22.12, 28.82)	25.39 (22.09, 28.69)	25.93 (19.34, 32.51)	
Less than high school	2.76 (1.91, 3.62)	2.90 (1.98, 3.82)	1.98 (0.58, 3.38)	
Marital status				<.0001
Married or living with partner	59.63 (53.63, 65.62)	57.09 (53.90, 60.28)	74.57 (69.25, 79.89)	
Other	40.37 (36.37, 44.37)	42.91 (39.72, 46.10)	25.43 (20.11, 30.75)	
Body mass index, kg/m^2^				.001
<18.5	2.19 (1.50, 2.89)	2.25 (1.52, 2.98)	1.87 (0.09, 3.65)	
18.5–24.9	34.15 (29.10, 39.20)	35.44 (32.01, 38.87)	26.52 (20.47, 32.57)	
25–29.9	23.61 (21.20, 26.03)	24.47 (22.38, 26.56)	18.57 (13.38, 23.76)	
≥30	40.05 (36.79, 43.30)	37.84 (35.33, 40.35)	53.05 (44.77, 61.32)	
Alcohol user				.17
Never	12.60 (9.77, 15.42)	13.19 (10.58, 15.79)	9.11 (5.19, 13.03)	
Former	4.71 (3.81, 5.62)	4.29 (3.27, 5.31)	7.20 (4.05, 10.35)	
Mild	26.74 (23.93, 29.55)	26.72 (23.91, 29.53)	26.83 (20.09, 33.57)	
Moderate	29.02 (24.97, 33.06)	29.15 (26.30, 31.99)	28.25 (22.27, 34.24)	
Heavy	26.94 (23.42, 30.45)	26.65 (23.95, 29.35)	28.61 (22.43, 34.79)	
Diabetes				.06
Diabetes	6.87 (5.81, 7.94)	6.31 (5.20, 7.42)	10.19 (7.45, 12.93)	
Impaired Fasting Glucose	2.85 (1.88, 3.83)	2.88 (1.93, 3.83)	2.69 (0.82, 4.55)	
Impaired Glucose Tolerance	2.09 (1.39, 2.78)	2.17 (1.40, 2.95)	1.56 (0.46, 2.67)	
No	88.19 (81.09, 95.29)	88.64 (87.09, 90.19)	85.56 (82.27, 88.85)	
Hypertension				<.001
No	84.60 (77.23, 91.96)	86.27 (84.46, 88.07)	74.76 (68.50, 81.01)	
Yes	15.40 (13.50, 17.31)	13.73 (11.93, 15.54)	25.24 (18.99, 31.50)	
Smoke				.25
Never	67.57 (62.12, 73.02)	68.40 (65.58, 71.21)	62.67 (57.09, 68.26)	
Former	12.65 (10.44, 14.85)	12.29 (10.28, 14.30)	14.73 (9.94, 19.53)	
Now	19.79 (16.89, 22.68)	19.31 (17.24, 21.38)	22.60 (16.36, 28.83)	
Ever treated for a pelvic infection/PID				<.001
No	95.52 (87.76, 103.27)	96.36 (95.43, 97.29)	90.54 (86.44, 94.63)	
Yes	4.48 (3.44, 5.52)	3.64 (2.71, 4.57)	9.46 (5.37, 13.56)	
Had regular periods in past 12 mo				.12
No	11.73 (9.91, 13.56)	11.20 (9.59, 12.81)	14.84 (10.40, 19.29)	
Yes	88.27 (81.25, 95.28)	88.80 (87.19, 90.41)	85.16 (80.71, 89.60)	
Ever taken birth control pills				.04
No	24.89 (22.48, 27.31)	25.80 (23.51, 28.09)	19.58 (14.10, 25.06)	
Yes	75.11 (67.93, 82.28)	74.20 (71.91, 76.49)	80.42 (74.94, 85.90)	
Age when first menstrual period occurred				.22
age < 10	3.83 (2.97, 4.69)	3.56 (2.73, 4.39)	5.42 (3.13, 7.72)	
10 ≤ age < 15	82.36 (75.89, 88.84)	82.41 (80.44, 84.38)	82.11 (77.51, 86.70)	
15 ≤ age ≤ 21	13.80 (11.49, 16.12)	14.03 (12.21, 15.85)	12.47 (8.22, 16.73)	
Uric acid, μmol/L	4.57 ± 0.03	4.53 ± 0.03	4.81 ± 0.08	.003
Triglycerides, mmol/L	1.32 ± 0.03	1.29 ± 0.04	1.46 ± 0.05	.02
HDL-cholesterol, mmol/L	57.19 ± 0.44	57.56 ± 0.45	55.02 ± 1.30	.07
Total cholesterol, mmol/L	4.66 ± 0.03	4.64 ± 0.03	4.80 ± 0.07	.02
Albumin, g/dL	4.18 ± 0.01	4.18 ± 0.01	4.16 ± 0.03	.31
Creatinine (µmol/L)	63.99 ± 0.52	63.96 ± 0.51	64.15 ± 0.97	.83
UHR (Box-Cox transformed)	−2.88 ± 0.01	−2.90 ± 0.02	−2.75 ± 0.04	.002
UHRQ				.01
Q1	25.72 (22.35, 29.08)	27.01 (24.19, 29.83)	18.07 (12.59, 23.55)	
Q2	26.72 (23.40, 30.05)	27.16 (24.43, 29.90)	24.15 (18.94, 29.35)	
Q3	24.05 (21.25, 26.84)	23.41 (21.05, 25.77)	27.79 (22.77, 32.81)	
Q4	23.51 (21.10, 25.92)	22.41 (20.18, 24.64)	29.99 (23.34, 36.64)	

HDL = high-density lipoprotein, UHR = uric acid-to-high-density lipoprotein-cholesterol ratio.

### 3.2. The association between UHR and infertility

**Figure 2.**
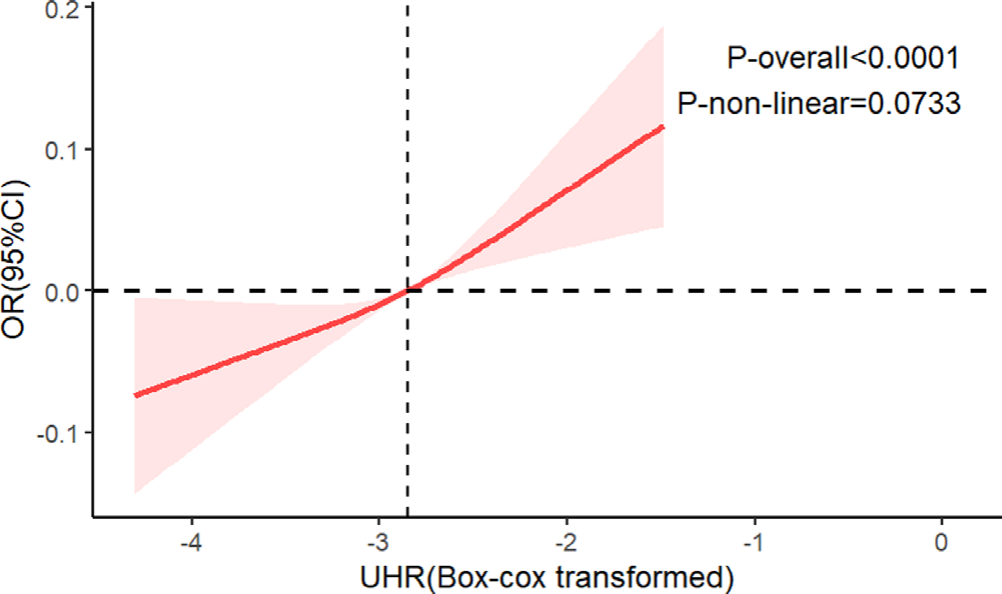
The smooth curve fit for the association between UHR and infertility. CI = confidence interval, OR = odds ratio, UHR = uric acid-to-high-density lipoprotein-cholesterol ratio.

Logistic regression was used to explore the association between UHR quartiles and infertility. As shown in Table 2, in the unadjusted model, UHR was positively correlated with infertility (OR = 1.735, 95% CI: 1.238–2.430). After adjusting for potential confounders, the association remained significant (model 1: OR = 1.823, 95% CI: 1.322–2.512; model 2: OR = 1.834, 95% CI: 1.244–2.702). Sensitivity analysis confirmed that UHR, when categorized into quartiles, maintained its association with the risk of infertility. Notably, participants in the highest quartile of UHR had a significantly higher risk (OR = 1.989, 95% CI: 1.147–3.452) compared to those in the lowest quartile. To further explore the relationship between UHR and infertility, smooth curve fitting was applied. Among all participants, a strong linear relationship between UHR and infertility was observed (Fig. 2).

**Table 2:** Logistic regression analysis of the association between the UHR with Box-Cox transformed and infertility risk.

Character	Crude model		Model 1		Model 2	
	95% CI	*P*	95% CI	*P*	95% CI	*P*
UHR (Box-Cox transformed)	1.735 (1.238, 2.430)	.002	1.823 (1.322, 2.512)	<.001	1.834 (1.244, 2.702)	.004
Q1	Ref		Ref		Ref	
Q2	1.329 (0.847, 2.085)	.210	1.464 (0.936, 2.291)	.093	1.536 (0.963, 2.451)	.069
Q3	1.774 (1.100, 2.862)	.020	1.889 (1.166, 3.059)	.011	1.896 (1.105, 3.255)	.023
Q4	2.000 (1.198, 3.338)	.009	2.106 (1.274, 3.480)	.005	1.989 (1.147, 3.452)	.018
*P* for trend		.005		.003		.014

Crudel model: UHRQ.

Model 1: UHRQ, age, and race.

Model 2: UHRQ, age, race, edu, pir3, tg, chol, alb, crea, alcohol.user, Diabetes, Hypertension, marital, and smoke.

UHR = uric acid-to-high-density lipoprotein-cholesterol ratio.

### 3.3. Subgroup analysis

**Figure 3.**
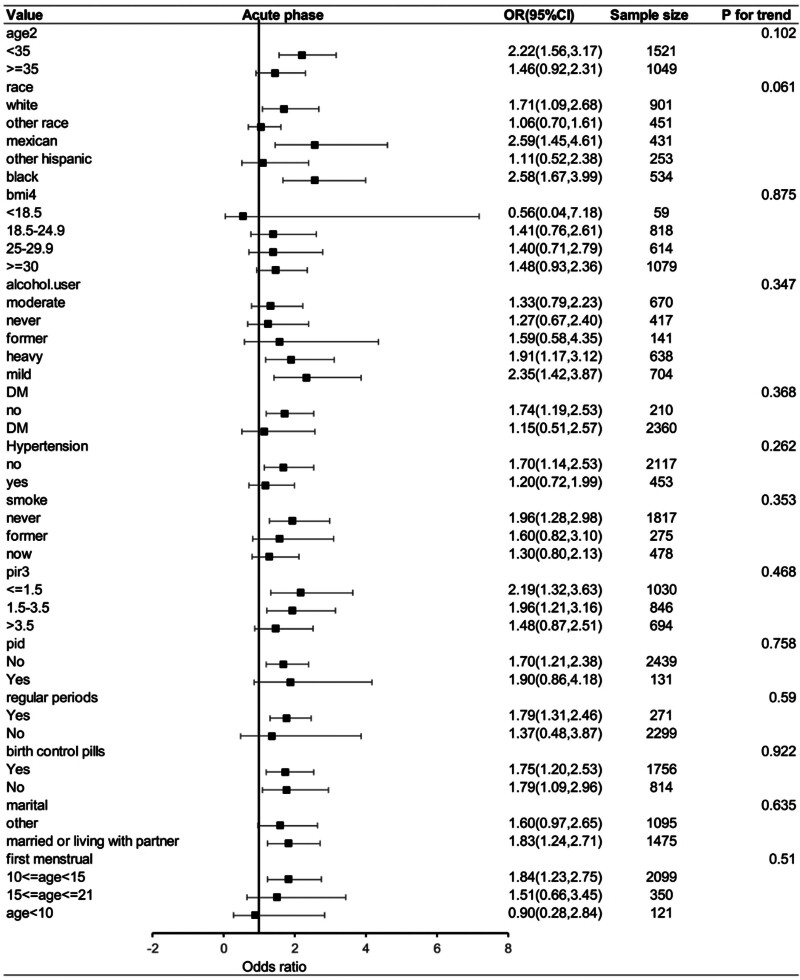
Forest plot of stratified analysis and interaction effects for the association between the UHR and infertility. CI = confidence interval, OR = odds ratio, UHR = uric acid-to-high-density lipoprotein-cholesterol ratio.

Figure 3 shows the results of the subgroup analysis for the association between UHR and infertility. The findings demonstrated that the effect of UHR on infertility was influenced by several factors, including age (<35 years), ethnicity (non-Hispanic whites, Mexican Americans, and non-Hispanic blacks), alcohol use (heavy/mild), diabetes status (no), hypertension status (no), smoke status (never), family poverty–income ratio (≤3.5), history of pelvic infection (no), regular menstrual periods in past 12 months (yes), use of birth control pills (yes/no), marital status, and age at first menstruation (10 ≤ age < 15). This indicates that the impact of UHR on infertility varied depending on these covariates.

### 3.4. The mediating role of BMI

**Figure 4.**
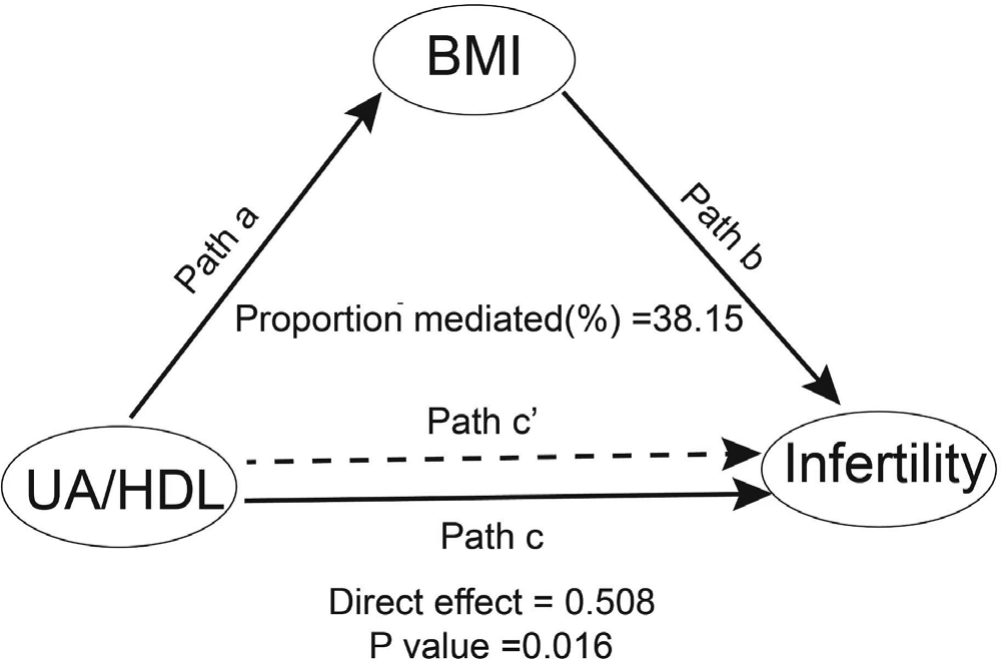
Mediated analysis model path diagram. Notes: UHR was defined as the independent variable; infertility as the dependent variable; and BMI as the mediating variable. Path a represents the regression coefficient of the association between UHR and BMI. Path b represents the regression coefficient of the association between BMI and infertility. Path c represents the direct effect of UHR on infertility when controlling for BMI. BMI = body mass index, UA = uric acid, UHR = uric acid-to-high-density lipoprotein-cholesterol ratio.

A causal mediation analysis was conducted to examine the potential mediating role of BMI in the association between UHR and female infertility. Figure 4 illustrates the mediation model and pathways, with UHR as the independent variable, BMI as the mediator, and infertility as the dependent variable. The results revealed that UHR exerted a significant indirect effect on the prevalence of female infertility through BMI, with an indirect effect size of 0.313 (95% CI: 0.118–0.510; *P* < .001). The mediation analysis demonstrated that BMI had a statistically significant partial mediating effect on the relationship between UHR and infertility. Moreover, after adjusting for BMI, the direct association between the UHR and infertility was no longer statistically significant (*P* = .06), indicating that BMI significantly influence the relationship between UHR and infertility through both direct and indirect pathways, with approximately 38.15% of the total effect mediated by BMI. The results of the mediation analysis, including direct effects, indirect effects, total effects, and the proportion mediated, are presented in Table 3.

**Table 3:** Mediation analysis of BMI in the association between UHR and infertility.

Independent variable	Mediator	Total effect coefficient (95% CI)	*P* value	Indirect effect coefficient (95% CI)	*P* value	Direct effect coefficient (95% CI)	*P* value	Proportion mediated (%)
UHR	BMI	0.82205 (0.473, 1.139)	<.001	0.31363 (0.118, 0.510)	<.001	0.50842 (0.109, 0.890)	.016	38.15

BMI = body mass index, UHR = uric acid-to-high-density lipoprotein-cholesterol ratio.

## 4. Discussion

This study examined the association between the UHR and female infertility using NHANES data and further explored the mediating role of BMI. We found that higher UHR levels were significantly associated with an increased risk of infertility among US women aged 18 to 45 years. Importantly, BMI partially mediated this association, accounting for approximately 38% of the total effect. These findings suggest that UHR may contribute to infertility both directly and indirectly through obesity-related metabolic pathways.

Our results are consistent with previous NHANES-based studies reporting that elevated UHR is positively correlated with female infertility.^[27]^ A prior analysis of 4171 women (NHANES 2013–2020) also identified a significant relationship between log-transformed UHR and infertility risk. Despite differences in transformation methods, both studies demonstrated a similar trend, reinforcing the robustness of this association. By employing Box-Cox transformation and incorporating mediation analysis, our study extends these findings by clarifying BMI’s partial mediating effect, which provides new insight into the biological pathways linking metabolic stress and reproductive health.

UA and HDL-C play opposing roles in oxidative and inflammatory processes.^[28]^ Elevated UA levels can increase OS and inflammatory activity,^[29,30]^ while HDL-C exerts antioxidant and anti-inflammatory effects. The UHR therefore reflects the balance between oxidative burden and antioxidant defense. Several studies have shown that higher UHR is associated with adverse metabolic conditions such as metabolic syndrome, diabetes, and chronic kidney disease.^[18,19,24–26]^ These disorders share underlying mechanisms involving inflammation, insulin resistance, and endothelial dysfunction, all of which can disrupt reproductive function.^[17,31]^ Our findings support the hypothesis that an elevated UHR increases oxidative and inflammatory stress, potentially impairing oocyte quality, follicular development, and endometrial receptivity.^[32,33]^

BMI was identified as a significant partial mediator in the relationship between UHR and infertility. Obesity exacerbates insulin resistance and hyperandrogenism, which disturb folliculogenesis and ovulation.^[34]^ In contrast, low BMI can suppress hypothalamic-pituitary-ovarian function, leading to anovulation.^[35,36]^ The observed partial mediation suggests that abnormal adiposity explains a substantial portion, but not all, of the effect of UHR on infertility.^[37]^ These findings highlight the intertwined roles of metabolic and inflammatory factors in reproductive dysfunction and emphasize the importance of maintaining optimal BMI in women of reproductive age. Given that UHR and BMI are both easily obtainable clinical parameters, their combined assessment may provide a practical approach for early identification of women at risk for infertility.

From a clinical perspective, monitoring UA and HDL-C levels together with BMI may help guide preconception counseling and preventive interventions. Weight reduction and lifestyle modification could mitigate UHR-associated risks by improving oxidative balance and metabolic function. Moreover, UHR may serve as a convenient composite biomarker for assessing both systemic OS and metabolic health in reproductive-age women.

Several limitations should be considered when interpreting these findings. First, due to the cross-sectional design, causality cannot be inferred between UHR, BMI, and infertility. Second, although major demographic and metabolic covariates were adjusted for, residual confounding from unmeasured variables cannot be completely excluded. Third, the analysis was limited to women aged 18 to 45 years in the United States, and the generalizability of the results to other populations remains to be established. Finally, BMI, while widely used, does not differentiate between fat and lean mass or reflect visceral adiposity, which may more closely relate to metabolic dysfunction. Future prospective studies incorporating central obesity measures and mechanistic biomarkers are warranted to confirm these findings and elucidate the underlying biological pathways.

## 5. Conclusion

This study provides evidence that a higher UHR is associated with increased infertility risk in US women and that BMI partially mediates this relationship. These findings underscore the importance of metabolic health in reproductive outcomes and suggest that the combined evaluation of UHR and BMI may offer a simple, cost-effective tool for early infertility risk stratification. Weight management and metabolic optimization could represent practical strategies to mitigate UHR-related reproductive risks. Further longitudinal research is needed to confirm causality and explore molecular mechanisms linking uric-lipid metabolism to reproductive dysfunction.

## Acknowledgments

The authors thank all participants and support staff who contributed to the NHANES.

## Author contributions

**Conceptualization:** Quan Yang, Meng-Hui Hong.

**Data curation:** Ming-xuan Du, Meng-Hui Hong.

**Investigation:** Lin Zhang.

**Methodology:** Meng-Hui Hong.

**Software:** Meng-Hui Hong.

**Writing – original draft:** Jia-Yi Zhao.

**Writing – review & editing:** Jia-Yi Zhao.
